# How Japan managed to curb the pandemic early on: Lessons learned from the first eight months of COVID-19

**DOI:** 10.7189/jogh.10.020390

**Published:** 2020-12

**Authors:** Urme Binte Sayeed, Ahmed Hossain

**Affiliations:** 1 Hiroshima University, Hiroshima, Japan; 2 Department of Public Health, North South University, Dhaka, Bangladesh; 3 Global Health Institute, North South University, Dhaka, Bangladesh

The outbreak of a severe respiratory disease caused by an unknown virus was first introduced in the Hubei province of China in late December of 2019. The virus's criteria were quite similar to the severe acute respiratory syndrome (SARS), which was occurred in 2003 but was less deadly and more dispersed than SARS. The virus was later named the 2019 novel coronavirus (COVID-19) by World Health Organization (WHO) and was declared a pandemic in March 2020 because of its widespread globally. According to official estimates, over 33 million people worldwide are contaminated, and almost a million have died until September 28, 2020. There is evidence of continuous transmission of the virus on six continents [1].

Japan is a developing nation with 127 million people residing in 364 555 km^2^; most of the residents live in urban areas (91.8%). The population in the country is 347 per km^2^. Tokyo is the capital city where 550 000 foreigners (6158 people per kilometer per square meter) reside. This means that Tokyo has a population of 2.4 times that of New York City. According to Japan's present age structure, 27.7% of the overall population are adults between the ages of 60-79 years, and 8.5% are 80 years and older [2].

Furthermore, the Japanese population's estimated life expectancy is 85.03 years. In 2016, Japanese men reached an excellent healthy life expectancy of 72.14 years and women 74.79 years [3]. Life expectancy is growing as health awareness is rising, and death rates are declining as the three leading causes of death in Japan include cancer, heart disease, and brain disease. This paper is intended to explain the factors behind Japan's success in curbing the virus in the early stages in comparison to other developed countries despite its being a “senior age” country.

## INCIDENCE PATTERN OF COVID-19 IN JAPAN

Japan was the third country to have the first COVID-19 case in January 2020 after Thailand. The country announced its first case on January 16, a returnee from Wuhan, China, and marked its first death case on February 13 due to Coronavirus, a woman in her 80s [3]. As of September 28, Japan has confirmed approximately 82 000 cases and 1545 deaths. A cursory view of the statistics may mean that Japan has had relatively moderate exposure to COVID-19 to date.

**Figure 1:**
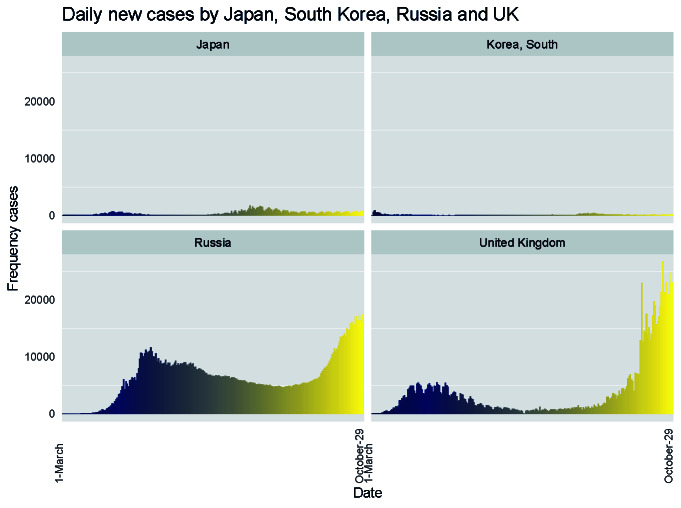
Daily new cases by Japan, South Korea, Russia and UK (data from JHU CSSE COVID-19 Dashboard).

**Figure 1** shows the incidence pattern of COVID-19 positive cases for the four peer countries of Japan. The incidence in Japan appears to be small compared to Russia and the United Kingdom. A short wave of infections seems to continue in Japan and South Korea. In the beginning, Japan's effective reproductive number was 1.38, and it had fallen <1, since early April when the US number was >1. Besides, it was calculated that approximately 80 percent of the infected people did not pass the infection to others.

## MORTALITY PATTERN OF COVID-19 IN JAPAN

**Figure 2:**
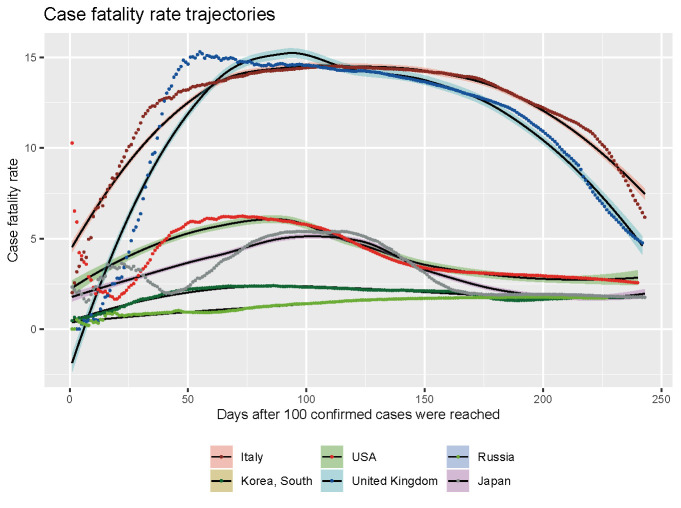
Case fatality rate trajectories of 6 countries by days after 100 confirmed cases.

With the virus spreading, it was soon found out that COVID-19 is an illness that threatens the elderly mainly, and Japan has more older adults per capita than any other country. The people of Japan are also thoroughly packed into huge cities. Despite all these, Japan faced few deaths from the beginning, along with only a 2.8% case fatality rate during the peak in April (**Figure 2**). In the case of the US, the case fatality rate was 14.5%, and in Italy, it was about 15% in the peak.

Japan and the Italian population vary from other countries in their demographic characteristics. In terms of morbidity and death due to COVID-19, the distribution of elderly inhabitants' ages from Japan and Italy is important. The infection in older patients is more lethal, and so the higher case fatality rate is observed both in Japan and Italy. In contrast to Japan, **Table 1** shows the age-specific deaths in Italy.

**Table 1:** Case fatality rate by age groups in Italy and Japan

	Japan as of August 26, 2020		Italy as of March 17, 2020	
**Age groups**	**No of deaths (%of total deaths) (known total = 1177)**	**CFR**	**No of deaths (% of total death) (total = 1625)**	**CFR**
0-9	0	0	0	0
10-19	0	0	0	0
20-29	1 (0.08)	0	0	0
30-39	4(0.34)	0.04	4 (0.3)	0.3
40-49	17 (1.44)	0.2	10 (0.6)	0.4
50-59	43 (3.65)	0.56	43 (2.7)	1
60-69	116 (9.86)	2.49	139 (8.6)	3.5
70-79	312 (26.51)	8.5	578 (35.6)	12.8
80+	684 (58.11)	21.3	850 (52.3)	20.2

*Case fatality rate (CFR) was calculated as number of deaths/number cases.

However, experts predicted that the primary SARS-CoV-2 virus circulating in most of Asia was the less-virulent B-type strain, although there is no strong evidence. In Japan, the most prevalent infected age is 20-29 years (22 833 cases), yet only two fatalities in this age was seen. On the other hand, Japan's most fatal age group is 80+ years (896 fatalities and 5093 cases as of September 30, 2020) [4].

## GOVERNMENT RESPONSES

The Japanese Government took relatively early steps to prevent the mass spread of the virus. The main concept of Japan was to maximize the suppression of the virus and to minimize the socio-economic damage of the country. Japan has a high standard health care system, which covered the medical care for COVID-19 under national Health Insurance with a high quality of medical care and local health centers even in rural areas. The pandemic was addressed by the Japanese Government using the following measures.

## DECLARATION OF NATIONAL EMERGENCY: JAPAN MODEL

After the initial report from China, Japan immediately revised its disease monitoring system to classify the suspected COVID-19 import cases. In the harbor of Yokohoma in Japan, the cruise ship *Diamond Princess* arrived with 3711 passengers and crew members on board, where a passenger disembarked from Hong Kong was tested positive earlier on February 1, and subsequently quarantined the entire ship for the next fourteen days. Later, 712 people (19.2%) were infected, and 13 died from the ship [5]. The response was then strengthened as the government demanded school closures; the government imposed restrictions on travel from China, South Korea, and other countries and revised health pandemic laws to include COVID-19 [5]. In late March, Tokyo's municipality advised people to stay home on weekends because of an upward increase in cases. On April 7, Prime Minister Shinzo Abe declared Tokyo and six other localities a national emergency, promoting social distancing and telework to reduce the virus's spread. This statement was revised on April 17 to encourage such acts nationally [6]. The Japanese version of COVID-19 lockdown is a little weaker than those enforced elsewhere, where the stay-at-home request was voluntary. When Prime Minister Shinzo Abe declared that he lifted the state of emergency on May 25, he proudly spoke about the *Japan Model*, suggesting other countries to learn from Japan.

## INTRODUCED RETROSPECTIVE CLUSTER-BASED APPROACH TO LOCATE TRANSMISSION

On February 25, the Ministry of Health, Labor, and Social Affairs, with the Government's aid, announced the Basic Policies for Novel Coronavirus Disease Control and set up a cluster response team along with 536 consultative centers [7]. The country used retrospective monitoring methods to find closer links to an infected person, while other countries employed the prospective approach to identify a major infection source. Japan's retrospective method was claimed to more reliably identify the initial source of infection and thus tracked all close contacts of sources of infection. The basic policy of the authority was to early detect the source of an infected individual through symptoms, follow all the people in the cluster who are highly transmissible, test and isolate them immediately and treat them as symptoms rather than general testing of the country's entire population [8]. The authorities succeeded in the cluster control approach in the earliest phase of the pandemic.

## AWARENESS PROGRAM WITH THREE Cs

At the beginning of the pandemic, the central Government of Japan asked people to avoid large gatherings, encouraged teleworking, and avoided unimportant trips, with the encouragement of avoidance of 3Cs. The places that meet the 3Cs are closed spaces, crowded places, and close-contact settings. Later Japan updated into “3C Plus” that included behavior modifications like avoiding loud talking and singing [9]. The national authority's policy was to raise public awareness and stop the spread of this virus without requiring a full shutdown.

## HIGH QUALITY HEALTH CARE SYSTEM

Japan was worried that hospitals were a source of cluster-infection due to overflowing patients in the hospital, putting the health care system to an enormous threat. The country coordinated hotels to prevent the hospital rush and asked the non-critical patients to stay at home or the designated hotels. The government also implemented robots to take care of patients in hotels and hospitals so that people can restrict their interactions with humans. Consequently, during the peak in April-May, only 17.1% of beds dedicated to COVID-19 were occupied by critical patients. [10]. There is no question that high-quality medical care, which is available to all Japanese people through universal health insurance, helps avoid the severity of illness.

## INTRODUCED CORONAVIRUS TRACING APP: COCOA

On June 19, the Government of Japan released a coronavirus tracing app for Android and IOS phones named “COCOA”. With the collaboration of Japan Microsoft, Apple, and Google, the app was launched to trace the people in contact with a COVID-19 positive patient [11].

## LIFESTYLE AND AWARENESS OF JAPANESE PEOPLE

According to experts, Japanese healthy lifestyles and distinct cultures, which vary significantly from the US or Europe, are the strength behind the early restraint. A recent report by the United States Centers for Disease Control and Prevention found people with underlying medical conditions such as heart disease, obesity, and diabetes are six times more likely to be hospitalized if they get COVID-19 and 12 times more likely to die. Compared to other developing nations, Japan has a low prevalence of co-morbidity, obesity, and diabetes due to its healthy eating habits and exercise habit [12]. The nutrition intake among Japanese people is well maintained, especially among the aged people who are most vulnerable to this disease.

**Figure:**
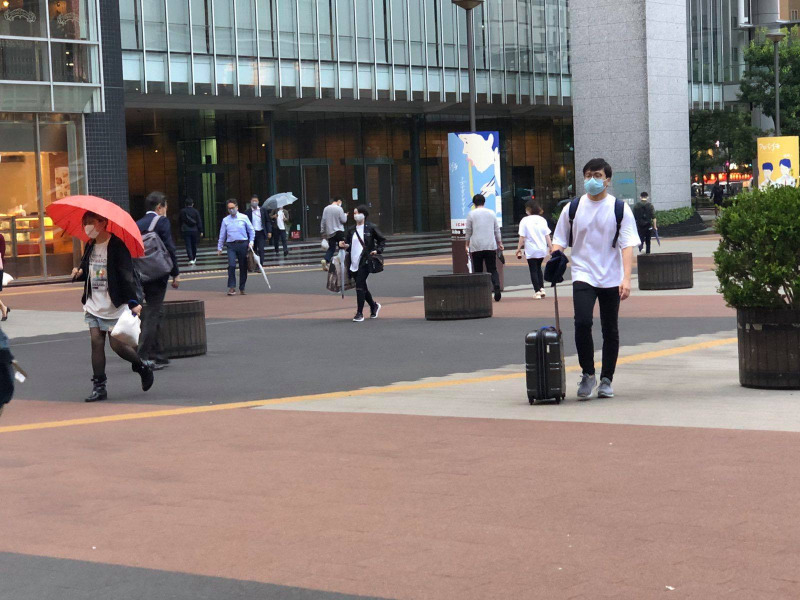
Photo: From Urme Binte Syeed’s own collection, used with permission.

Japan's mild lockdowns seem to have had a real lockdown effect. While people were not forced to remain at home, they did in general. The citizens of Japan have compiled with authority from the very beginning of the pandemic. As a result, the crowds dropped significantly in most of the prefectures. For instance, when people often visit within and outside the country during “Golden Week” holidays, the governors of the localities asked people to stay home and avoid unnecessary movements. As a result, the people kept away from mass gatherings and trivial trips in compliance with the request.

There is a recent hypothesis that most COVID-19 affected countries are severely vitamin D deficient. For example, Europe is the most infected area of the world, where approximately 40% of people are moderately, and 13% are severely vitamin D deficient [13]. Comparative to that among Japanese people, the prevalence of hypovitaminosis D is less than 5% among active older adults and 10.3% among women over 30 years that is very low that might be a contributing factor for the success of the country. Moreover, several scientists anticipated that the countries without a universal BCG vaccination policy might witness a more devastating situation. Thus, the US, Netherlands, and Italy got many critical infections. A report suggested that the fatality rate of COVID-19 among the countries with the BCG vaccine is 4.1%, where it is 5.1% among the countries without the vaccine [14]. Japan started its universal and compulsory BCG vaccine in 1947, which helped fewer deaths per million people than the US and Italy [15].

Furthermore, the exchange of greetings between Japan and the rest of the world varies. In greeting, the Japanese tend to bow rather than shake, embrace, or kiss. Japanese people were believed to have droplets smaller than others; it was shown that approximately 80% of patients did not transmit this virus to others. Also, an established culture of masks, especially in the winter grip season, maybe an important reason for the low infection. Furthermore, in Japan, which is practiced widely in educational institutions from a very young age, a tradition of handwashing is higher.

## A SECOND WAVE IN JAPAN?

After producing the first COVID-19 wave, the country gained global attention with Japan Model – restricted testing and no lock-up, nor regulatory means to force businesses to shut down. However, the rise in new cases in Japan was alarming in July. The upward trend in July gave rise to Tokyo's fears of an imminent second wave. Although the infection rate rose in July, the Government of Japan took no measures to reintroduce the state of emergency or restrict travel across prefectural borders. After most of May spent on negative territories, the national reproductive index rose to 1.8 on July 6. One of the highlights is that the test positivity rate stays below 4.0%. Though the capital has seen an increase since July, the number of cases has declined during September (**Figure 1**). In view of a steady decline in new cases within Tokyo, in mid-September, the government withdrew the shortened business hours that were introduced at the end of July [16]. So, Japan has managed to control the virus until today.

## CRITICISM AND CONTROVERSIES

Critics said that the Premiers voluntarily kept a low testing rate because of the upcoming Olympics in 2020 and a fear of a possible collapse in medical facilities [17]. The test rate of the country was shallow than in other peer countries. To track the outbreak, the South Korean government launched a major test program, but Japan did the reverse. Japan worried that people who tested positive but only had mild symptoms would be going to hospitals. However, the government in Japan relied on the public to support them. Japan asked people to look after themselves, stay away from crowded areas, wear masks and wash their hands, and by and large, that is precisely what most people have done.

## CONTRIBUTION TO COVID-19 VACCINE

To combat the war against the Coronavirus, the research institutions and pharmaceutical companies worldwide have begun a competition to develop a vaccine against the COVID-19. According to WHO, 142 candidates are working on manufacturing the vaccine, of which 13 candidates begun their clinical trials before the end of March. The vaccines from the US, UK, and China are now in the third phase. In this race, Japan is not far behind. A Japanese pharmaceutical company named Daiichi Sankyo has collaborated with the University of Tokyo to generate COVID-19 vaccines, which is supported financially by the Japan Agency for Medical Research and Development (AMED). Besides, a Japanese Biotech Company AnGes, has entered into a partnership with Osaka University along with the National Institute of Infectious Diseases that is observed to be the front contestant in developing a DNA vaccine. The DNA vaccine protocol is that an engineered circular DNA will be pushed inside a human body that will produce “spike proteins” inside the body. When the proteins are made inside the body, the body’s immune system will be invigorated and produce antibodies against it. The Japanese Government is willing to aid the domestic developments of vaccine production. The Japan Agency for Medical Research and Development has announced distributing 10 billion Yen (US$93.5 million) among nine projects [18]. Prime Minister Shinzo Abe said on August 28; Japan would secure COVID-19 vaccinations for all citizens by the first half of 2021.

## LESSONS LEARNED

What happened in Japan shows that the spread of COVID-19 can be controlled in quarantine, social distancing, and isolation of infected people. The key lesson from Japan is that while the country succeeds in containing for the first time, isolating and sustaining containments is difficult for a long time. It is challenging to identify community transmission and hospital transmission unless a country increases its testing capability. The next lesson is that the “new reality” will take much longer than anyone anticipated. Another lesson is that even though a country succeeds in containment locally, it is impossible to retain a virus-free status in other regions of the country, as long as people move around.

In contrast to other countries, Japan has never talked about eliminating the pathogen. Speaking of the current time, Japan's experts tried to promote a “new way of living” where people will have to deal with the virus. Japan followed the idea of maximizing efforts to suppress transmission and to minimize socio-economic damage. Ideally following three preventive steps can help a country to contain the epidemic:

1. Early detection of cluster and early response,
2. Enhancement of intensive care and securing medical service system for the severely ill patients, including medical equipment (Ventilator, ECMO, etc),
3. Behavior modification of citizens.

## CONCLUSION

Science has shown that Japan has, so far, controlled the dissemination of the disease. Telecom giant Softbank conducted an antibody test on 40000 employees in June that revealed only 0.24% were exposed to the virus. Randomized studies in Tokyo and two other prefectures found that exposure rates were much lower. In Tokyo, only 0.1% came back positive. As opposed to rigorous lockdown measurements and widespread testing like in other countries, it is only with the help of pre-existing cultures and healthy lifestyles that Japan has successfully reduced the transmission of SARS-CoV-2. Implementing an instant breakdown of smaller clusters by retrospectively tracing and motivating a 3C method for all the people appeared to be successful. This study could help many countries address many aspects of the successful management of infections.
